# An Evaluation of the Efficacy and Acceptability of Basal Implants in Traumatically Deficient Ridges of the Maxilla and the Mandible

**DOI:** 10.7759/cureus.43443

**Published:** 2023-08-13

**Authors:** Geeti Vajdi Mitra, Nikit Agrawal, Nupur Shukla, K. Aishwarya, Ponnamma C.C, Amal Raj

**Affiliations:** 1 Department of Oral and Maxillofacial Surgery, Sri Aurobindo College of Dentistry, Indore, IND; 2 Department of Oral and Maxillofacial Surgery, Sri Aurobindo Institute of Dental Science, Indore, IND; 3 Dentistry, Government Dental College and Hospital, Vijayawada, IND; 4 Department of Oral and Maxillofacial Surgery, ESIC Dental College, Kalaburagi, IND; 5 Department of Oral and Maxillofacial Surgery, Malabar Dental College and Research Centre, Manoor, IND

**Keywords:** implant dentistry, atrophic ridges, edentulous maxillary and mandibular arches, basal implants, implants

## Abstract

Background and objective

Rehabilitation of edentulous jaw areas is a complex procedure that has witnessed numerous advancements in technique and materials for better functional and aesthetic outcomes over the years. Dental implants have emerged as a cutting-edge, cost-effective, and non-invasive alternative to traditional removable partial dentures (RPDs), fixed partial dentures (FPDs), and full dentures. In this study, the basal implant was tested in traumatically deficient ridges on the mandible and the maxilla to determine its effectiveness and acceptability.

Materials and methods

The purpose of this research was to determine whether basal implants might be successfully used to restore traumatized ridges in the maxilla and mandible. Eleven individuals aged 20-55 years participated in the trial, and a total of 30 implants were used. All patients were evaluated for pain, soft tissue health around the basal implant, and patient satisfaction, with follow-ups on the third day and at three and six months postoperatively.

Results

The mean pain score measured using the visual analog scale (VAS) of patients during follow-ups was 2.00 ±1.15 on the third day, 0.40 ±0.84 at three months, and 0.00 ±0.00 at six months postoperatively. The mean gingival index score of patients was 1.30 ±0.48 on the third day, 0.10 ±0.31 at three months, and 0.00 ±0.00 at six months postoperatively. The mean patient satisfaction score was 34.77±5.54 on the third day, 41.11 ±6.27 at three months, and 40.7 7±10.91 at six months postoperatively. The mean immediate postoperative marginal bone level was 12.33 ±2.26 mm, and it was 10.85 ±2.29 mm after six months postoperatively. The mean preoperative crestal bone level was 10.76 ±1.77 mm and it was 9.70 ±1.79 mm after six months postoperatively.

Conclusion

Due to the fact that basal implantology does not involve extensive augmentation procedures necessary for the placement of conventional implants, it plays an important role in the rehabilitation of edentulous maxillary and mandibular arches, especially in traumatic, moderate to severely atrophic maxillary and mandibular arches.

## Introduction

The rehabilitation of edentulous areas of the jaw is a complex procedure, and various innovations in the technique and materials have emerged over the years for better functional and aesthetic outcomes. Removable partial dentures (RPDs), fixed partial dentures (FPDs), and complete dentures are highly cost-effective treatment options available in the wake of recent advances, pertaining to dental implants for the rehabilitation of partially and completely edentulous ridges.

Since conventional implants are placed in the crestal alveolar bone, they may require a secondary surgical procedure before implant placement [1]. Patients presenting with severely traumatized jawbones can be treated with a basal implant, which derives its support from basal bone [2]. Restoring the function and signature esthetics of the masticatory device in complex or extremely complex anatomic situations with minimally invasive techniques that adhere to standard biological, anatomical, physiological, and mechanical norms while giving due consideration to hygienic and esthetic requirements is the primary goal of the field of basal implantology [3].

The idea of basal implant loading has become widely popular and is being adopted by several dentists and patients as it is associated with less trauma, shorter treatment times, less hard and soft tissue resorption, better function and esthetics, and greater patient acceptance because its main anchorage is by cortical engagement [4]. This study aimed to assess how well the basal implant blended with the traumatically compromised ridges.

## Materials and methods

Patients of both genders aged 20-55 years participated in this research, which was carried out at the Oral and Maxillofacial Surgery Outpatient Clinic at the Sri Aurobindo College of Dentistry in Indore. Ethical approval was obtained from the Institutional Ethical Committee with the reference number: IEC/OS/PG/22. The study evaluated treatment outcomes in patients with deficient alveolar ridges secondary to trauma or surgery, which were indicated for basal implants. Parameters such as pain, soft tissue health, changes in bone level, and patient satisfaction were evaluated postoperatively on the third day and at three and six months. A total of 30 implants in 11 patients who were partially edentulous were studied.

Patients with systemic illnesses or limiting conditions that excluded surgery, non-attendees, and those with bruxism, clenching, mouth breathing, mental problems, temporomandibular joint issue, or drug misuse were not included in the research. All individuals provided informed consent before participating in the research.

Preoperatively, individuals who lacked adequate alveolar ridges were considered. Preoperative clinical evaluation included both medical and dental histories and a history of drug allergy. Complete intraoral and extraoral clinical examinations were done to access any abnormality. All patients were advised to undergo preoperative panoramic radiographs and cone-beam CT (CBCT) for ridge evaluation including the height and width of the residual alveolar ridge. A customized surgical stent was fabricated for each patient to guide the proper angulation and position of the basal implants. The armamentarium included a physio-dispenser with handpiece, Simpladent Ihde dental starter surgical kit (Simpladent, Ghaziabad, India), and a basal dental implant (BCS) by Simpladent.

**Figure 1:**
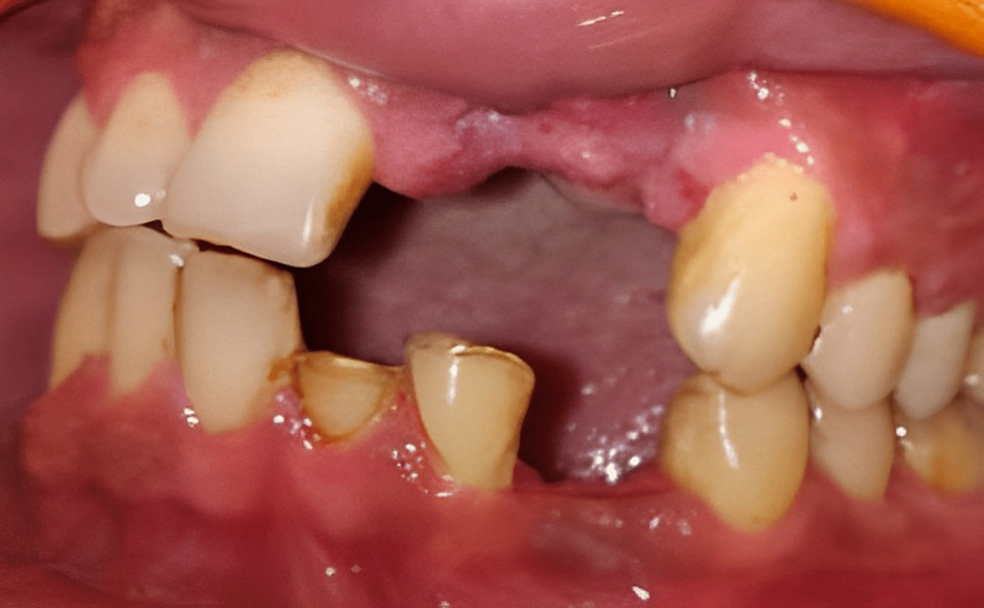
Preoperative intraoral photograph

**Figure 2:**
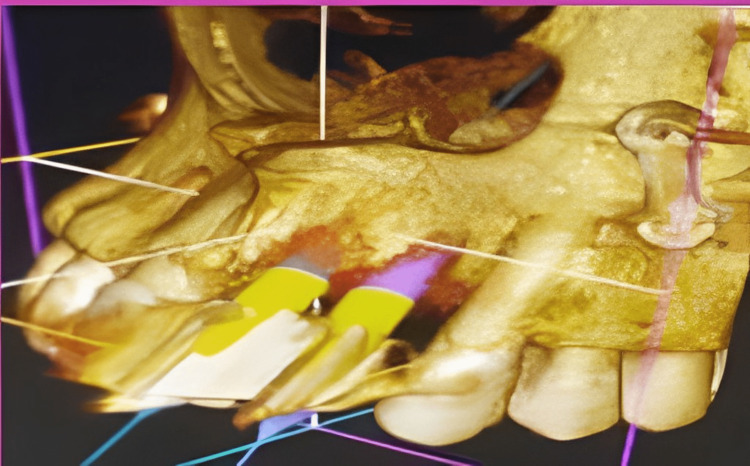
CBCT image of treatment planning CBCT: cone-beam computed tomography

**Figure 3:**
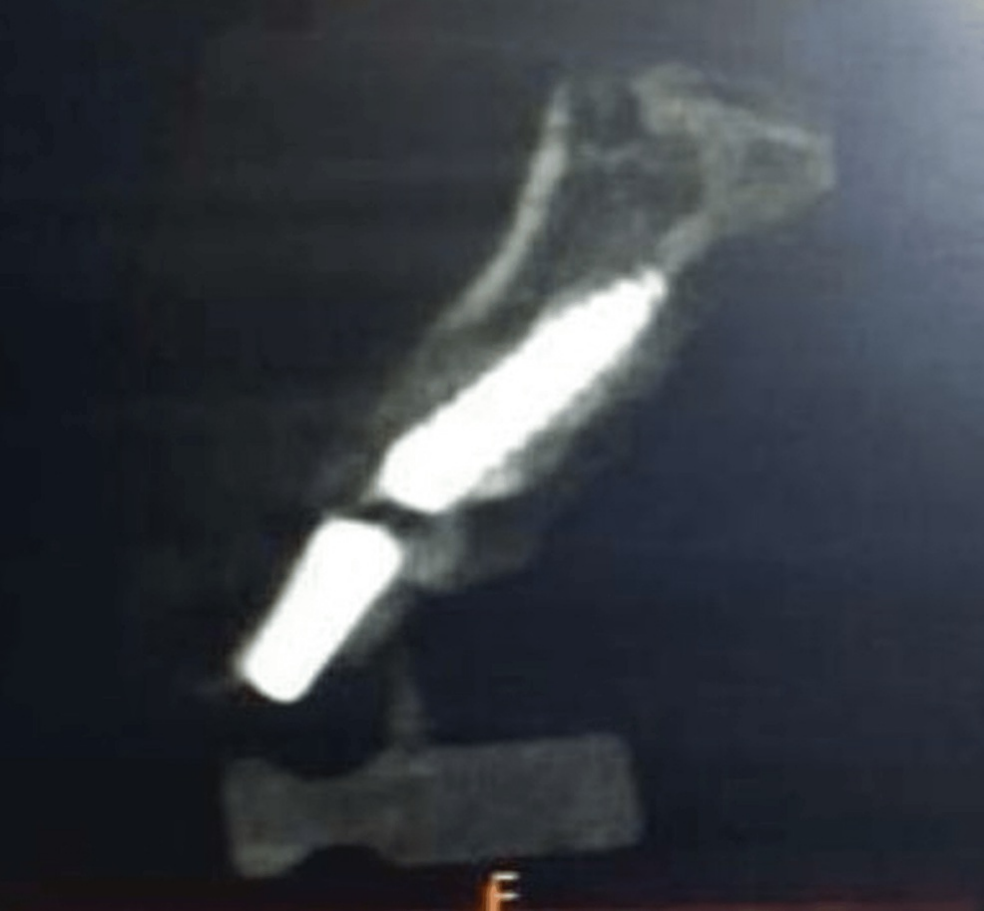
Postoperative scan image at six months

All of the procedures were performed with lignocaine (2% adrenaline, 1:200,000) as a local anesthetic. The CBCT evaluation helped determine the preoperative length of the basal implant. Using a 2-mm route-finding drill and a bespoke surgical guide placed on the ridge, the first cortex (alveolar) was drilled to confirm the ideal location, orientation, and angle for engaging the cortical bone. Drilling proceeded with either DOS-1 or DOS-2 (KOC design of basal implant) or BCD-1 to BCD-2 (BCS design of basal implant), until the basal cortical bone was reached around the nasal floor/sinus floor/lingual cortex/pterygoid bone, which was experienced as a dip. The implant was placed and screwed in until it engaged the bone at its most superficial layer (the basal cortical bone), which required a torque of 30 or more. After an implant was placed, any necessary bending was done to the implant's neck to ensure correct alignment and subsequent prosthetic rehabilitation. In the first radiograph taken after the procedure, the site of the basal implant was verified. Putting on the impression caps and making the imprint, the metal trial was sent to the prosthetics lab. The patient's occlusal records/jaw relations were noted, and a metal try-in was performed on day two. On day three, occlusal modifications were made if necessary, and the finished prosthesis was cemented into place using GIC luting cement (Figures 1-3).

Patients were instructed to take oral antibiotics [amoxicillin 500 mg + clavulanic acid 125 mg (625mg)] every 12 hours for five days and non-steroidal anti-inflammatory analgesics (diclofenac potassium 50 mg) every eight hours for five days after surgery, as well as to apply cold fomentation intermittently and avoid rinsing or drinking hot beverages for 24 hours.

Pain, soreness, and other indicators of discomfort were assessed in every patient. The discomfort was measured by means of a visual analog scale (VAS) [5]. Patients were asked to describe their level of discomfort on a scale from 0 (no pain) to 10 (extreme pain) [6]. CBCT was employed to promptly examine all implants both initially and six months later. The linear measures from CBCT images were used to assess the changes in mesial and distal bone height around the implants. Alveolar crest level was measured from the abutment tip to the implant-bone contact. The two sides were averaged out. Both immediately after surgery and six months later, the marginal bone level was measured and compared for analysis.

To compare the preoperative and the postoperative results of change in the crestal bone level, the occlusal level was considered as a reference point of the adjacent teeth to the ridge where the implant had to be placed and was measured. The change in crestal bone level was calculated with the help of CBCT and preoperative levels were compared with those at six months postoperatively. Patient satisfaction was evaluated to determine the efficiency of basal implants in terms of functional and esthetic outcomes, by utilizing a questionnaire involving categorized statements. The responses to statements were recorded according to the Likert response scale, as follows: 5 = strongly agree; 4 = agree; 3 = neither agree nor disagree; 2 = disagree; and 1 = strongly disagree.

We used IBM SPSS Statistics version 20.0 (IBM Corp., Armonk, NY) to do some basic statistical analyses on the data that we gathered and then plugged it into an Excel spreadsheet for easy viewing. A p-value of 0.05 was considered statistically significant, and the significance threshold was set at 5%. To ensure that the data were normally distributed, the Kolmogorov-Smirnov and Shapiro-Wilks tests were used. Analyses of quantitative variables were conducted using the student's t-test, Mann-Whitney U-test, analysis of variance (ANOVA), and post hoc analysis.

## Results

**Figure 4:**
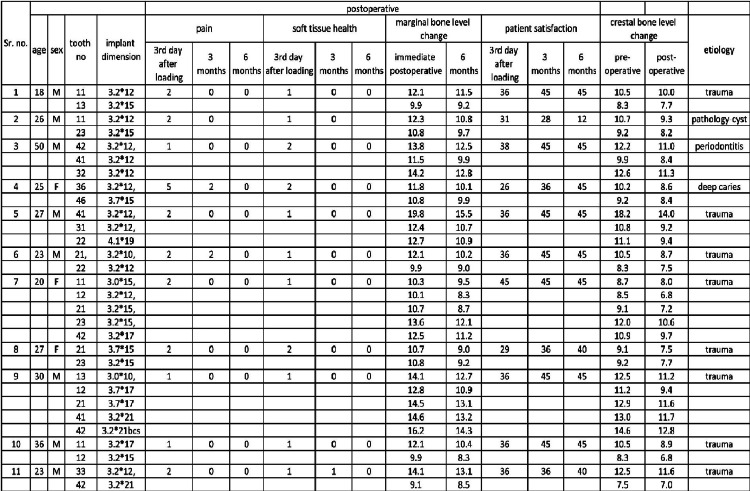
Master chart of the parameter values

A total of 30 implants were put in 11 participants aged 20-55 years for the trial. The mean age of the patient was 27.72 ±8.83 years. All patients were evaluated for pain, soft tissue health around the basal implant, and patient satisfaction on the third day and at three and six months postoperatively. The marginal bone level change was compared by taking radiographs preoperatively and at six months postoperatively. All the patients had reported on the third day and at three and six months after the procedure. The master chart of the values of each parameter is presented in Figure 4.

The pain was measured by using a VAS ranging from 0 to 10. The mean pain score was 2.00 ±1.15 on the third day, 0.40 ±0.84 at three months, and 0.00 ±0.00 at six months postoperatively. Repeated measures ANOVA test showed a significant difference between the scores with an F value of 24.38 and a p-value of 0.001. A pairwise comparison of pain between different time periods was done by using the post hoc Bonferroni test, which showed a significant reduction of pain between the third day and third month and the third day and six months; however, the difference between pain scores at three months and six months was not significant (Table 1).

**Table 1:** Pain scores at different time periods SD: standard deviation

Time period	N	Mean ±SD	F value	P-value
Third day	11	2.00 ±1.15	24.38	0.001
Third month	11	0.40 ±0.84		
Sixth month	11	0.00 ±0.00		

The soft tissue health was measured by using the Loe and Silness gingival index, ranging from 0 to 3. The mean gingival index score of patients was 1.30 ±0.48 on the third day, 0.10 ±0.31 at three months, and 0.00 ±0.00 at six months postoperatively. Repeated measures ANOVA test showed a significant difference between the scores with an F value of 42.81 and a p-value of 0.001. A pairwise comparison of soft tissue health was done between different time periods by using the post hoc Bonferroni test, which showed significant improvement in soft tissue health between the third day and third month and the third day and sixth month; however, the difference between soft tissue health at three months and six months was not significant (Table 2).

**Table 2:** Soft tissue health at different time periods SD: standard deviation

Time period	N	Mean ±SD	F value	P-value
Third day	11	1.30 ±0.48	42.81	0.001
Third month	11	0.10 ±0.31		
Sixth month	11	0.00 ±0.00		

Patient satisfaction was evaluated by using a questionnaire, which involved a Likert response scale, with scores as follows: 5 = strongly agree; 4 = agree; 3 = neither agree nor disagree; 2 = disagree; and 1 = strongly agree, which means that the patients who were completely satisfied with the outcome should have a total score of 45 and those who were not satisfied completely should have a score of 9. The mean gingival index score of patients was 34.77 ±5.54 on the third day, 41.11 ±6.27 at three months, and 40.77 ±10.91 at six months postoperatively. Repeated measures ANOVA test showed a significant difference between the scores with an F value of 3.88 and a p-value of 0.042. The decrease in patient satisfaction score was mainly due to the failure of the implant in two patients. Pairwise comparison between different time periods was done by using the post hoc Bonferroni test, which showed a significant difference in patient satisfaction between the third day and third month and the third day and sixth month; however, the difference between patient satisfaction at three months and six months was not significant (Table 3).

**Table 3:** Patient satisfaction at different time periods SD: standard deviation

Time period	N	Mean ±SD	F value	P-value
Third day	11	34.77 ±5.54	3.88	0.042
Third month	11	41.11 ±6.27		
Sixth month	11	40.77 ±10.91		

CBCT was taken immediately after surgery and again six months later to quantify linear changes in bone height surrounding the basal implants. This was done to determine the level of the marginal bone. The immediate postoperative mean marginal mone level was 12.33 ±2.26 mm, and it was 10.85 ±2.29 mm after six months of surgery. Paired t-test showed a significant difference between the scores with a t value of 5.25 and a p-value of 0.001 (Table 4).

**Table 4:** Marginal bone level before and after the treatment SD: standard deviation

Time period	N	Mean ±SD	Change in bone level	T value	P-value
Before treatment	30	12.33 ±2.26 mm	1.48 ±0.38 mm	5.25	0.001
Sixth month	30	10.85 ±2.29 mm			

Crestal bone level was evaluated by comparing CBCT scans preoperatively and after six months postoperatively. The mean crestal bone level preoperatively was 10.76 ±1.77 mm and it was 9.70 ±1.79 mm after six months. Paired t-test showed a significant difference between the scores with a t value of 3.29 and a p-value of 0.001 (Table 5).

**Table 5:** Crestal bone level before and after the treatment SD: standard deviation

Time period	N	Mean ±SD	Change in bone level	T value	P-value
Before treatment	30	10.76 ±1.77 mm	1.06 ±0.30 mm	3.29	0.001
Sixth month	30	9.70 ±1.79 mm			

## Discussion

The primary goals of developing basal implants like BOI®, Diskos®, KOC, and BCS are for immediate usage and for use in the atrophying jawbone. Furthermore, they may be positioned in the sinus area, where horizontal bone is abundant but vertical bone is lacking. Today's basal implants have a smooth, polished surface since it was discovered that smooth surfaces are less likely to cause inflammation (mucositis, periimplantitis) compared to rough ones. The basal Implants are placed in the lower part of the jaw where there is less risk of bone resorption and infection [7].

Without a surgical guide or stent, there can be a change of angulation or 1-2 mm of discrepancy in the point of insertion of the basal implant, which is decided by the preoperative CBCT. Hence, while performing our study, image-guided placement of basal implants using a prefabricated surgical stent was performed with the help of CBCT. The patient’s preoperative impression was taken and, with the help of CBCT the reference point of insertion of the basal implant was marked on the cast; this point of insertion was decided by the angulation through which we can achieve cortical engagement. This research set out to assess the usefulness and acceptability of basal implants for restoring missing teeth in the maxilla and mandible. Eleven patients aged 20-55 years received a total of 30 implants throughout the course of the trial. All patients were evaluated for pain, soft tissue health around the basal implant, and patient satisfaction on the third day and at three and six months postoperatively. The marginal bone level change was compared by taking radiographs/CBCT preoperatively and after six months postoperatively.

The mean pain score was 2.00 ±1.15 on the third day, 0.40 ±0.84 at three months, and 0.00 ±0.00 at six months postoperatively. The test result showed a significant reduction in pain from the third day to the third month. This is because the incidence of peri-implantitis is much reduced owing to the smooth surface designs of basal dental implants, which prevent bacterial colonization on the implant surfaces [8]. Our findings are in line with those of Omar et al. [9] as most of the patients experienced mild pain (VAS = 1) and moderate pain (VAS = 2) for a duration of one to three days after surgery.

The mean soft tissue health in the study as per the gingival index score was found to be 1.30 ±0.48 on the third day, 0.10 ±0.31 at three months, and 0.00 ±0.00 at six months postoperatively. The test result showed significant improvement in soft tissue health from the third day to the third month. Basal implants and abutments are deemed to be biologically inert, with no adverse or unfavorable reaction with host tissues, due to which there is reduced gingival inflammation and hence a reduction in gingival index score [10]. Anuradha et al. [11] found that the sulcular bleeding index value at baseline was 1.92 ±0.29, which decreased to 1.49 ±0.28 at one month and to 1.107 ±0.25 at the end of three months postoperatively. In our study, the average marginal bone loss was 1.480 ±38 mm, whereas the average crestal bone loss was 1.060 ±30 mm. Marginal bone loss in the maxilla was 1.740 ±37 mm, and in the mandible, it was 1.230 ±35 mm between preoperatively and six months postoperatively. Consequently, the marginal bone loss in the maxilla exceeded that in the mandible. Loss of crestal bone was 1.210 ±15 mm in the maxilla and 0.910 ±21 mm in the mandible on average, which shows that the loss of crestal bone in the maxilla was also more severe than that in the mandible.

Using the Galileos CBCT software, Gaber et al. [12] showed the marginal bone level, assessed the average bone height surrounding the basal implants immediately after surgery, and found that it rose from 7.3 mm to 7.7 mm over the course of three months. The current study observed that three out of 30 implants failed after three months and the survival rate for basal implants was 90.67%. After an average of 18.93 +8.41 months of monitoring, Oleg et al. [13] found a very high cumulative implant survival rate of 95.7%. In comparison to crestal implants, proponents of basal Implant systems claim that they can repair almost every situation with greater success in less time and with a lower risk of complications [14,15]. These findings, however, are still being tested over the long term.

This study has a few limitations, including the short follow-up period and the limited sample size. Clinical decision-making and treatment outcomes may both benefit from future research with larger sample sizes and longer follow-up periods. Also, a more precise methodology of placement of the basal implants by improved surgical stents can provide much better outcomes.

## Conclusions

With benefits including a flapless technique that needs little to no surgery, less pain after surgery, less swelling, and reduced discomfort, basal implantology is a novel therapeutic procedure with new, wide indications. Since basal implants do not need prolonged augmentation operations unlike traditional root-form implants and permit quick loading, it is reasonable to conclude that they may play an important role in repairing atrophying jaws with diminished bone quality or quantity. However, a more precise methodology of placement of the basal implants by improved surgical stents can provide much better outcomes.
